# Accounting for data sparsity when forming spatially coherent zones

**DOI:** 10.1016/j.apm.2019.03.030

**Published:** 2019-08

**Authors:** Kirsty L. Hassall, Andrew P. Whitmore, Alice E. Milne

**Affiliations:** a Computational and Analytical Sciences, Rothamsted Research, Harpenden, AL5 2JQ, UK; b Sustainable Agricultural Systems, Rothamsted Research, Harpenden, AL5 2JQ, UK

**Keywords:** Precision agriculture, Spatial coherence, Data sparsity, Cluster analysis, Crop yields

## Abstract

Efficient farm management can be aided by the identification of zones in the landscape. These zones can be informed from different measured variables by ensuring a sense of spatial coherence. Forming spatially coherent zones is an established method in the literature, but has been found to perform poorly when data are sparse. In this paper, we describe the different types of data sparsity and investigate how this impacts the performance of established methods. We introduce a set of methodological advances that address these shortcomings to provide a method for forming spatially coherent zones under data sparsity.

## Highlights

• There exist three distinct types of data sparsity that inhibit the formation of spatially coherent zones.
• By defining a neighbourhood of points through the Voronoi partition, the effects of spatial sparsity can be avoided.
• Observed data points are often not co-located, resulting in big data losses through current methods.
• By implementing missing data approaches within the clustering algorithms, data loss can be mitigated.

## 1 Introduction

It is a well-recognised aim of many on-farm management strategies to divide fields into zones to ensure efficient and effective management where each zone may be treated differently. Defining such zones has been a topic of research for at least 40 years (see e.g. [1]). The process of defining zones depends upon both the variables used to inform the zones, but also the approach used to ensure the zones are spatially coherent. It is of limited practical use to farm management if resulting zones are small and disjointed [2].

Data used to inform zones most commonly include yield data or soil characteristics which can be measured either directly or more recently via remote sensing [3], [4], [5], [6].

Once data are gathered and processed appropriately, methods for forming spatially coherent zones generally consist of two steps; clustering and smoothing. However, the literature varies in both the order that these steps are taken and the specific clustering and smoothing methods used. For instance, [7] and [8] induced spatial smoothing through a modified dissimilarity/similarity matrix based on the variogram/covariance between points that was then used in the subsequent clustering. In comparison, [9] first classified the data through fuzzy c-means clustering and then smoothed the resulting clusters. This method was shown to outperform [7] and [8] in [2]. There are also implementations where data are first smoothed (e.g. through kriging) and are then classified [3], [4], [6]. Despite the lack of consensus both in the choice of smoothing method and also in how the smoothing is implemented, there has been a (somewhat) linear evolution in the approach to classification. Specifically, early work used hierarchical clustering methods [1], however, since soil is not intrinsically hierarchically structured [2], and the advancement of computational power, non-hierarchical methods such as k-means became feasible [7], [8]. Furthermore, since the late 1990s, non-hierarchical “fuzzy” clustering approaches have been prevalent in the literature [2], [3], [5], [6], [9]. Fuzzy c-means was first developed by [10] and assigns each point to a cluster with a specified probability. This then allows one to see which points are well distinguished and which are “fuzzy”.

In this work, we return to the approach of [9] and [2], but look to address the specific issues associated with data sparsity. Data sparsity can impact a dataset in different ways, through *variable sparsity, spatial sparsity* or *colocation sparsity*.

Variable sparsity refers to a lack of information in the set of measured variables. Yield data often exhibit a high level of variability across time and space. Thus, to be able to definitively identify distinct clusters, several years worth of data need to be collected in order to look for consistently high or consistently low yielding areas. However, if the yield data are variable sparse and contain too little information, i.e. that the signal is too weak compared to the variability, clusters will be difficult to identify and distinguish regardless of how many years’ data are available.

Spatial sparsity occurs when data are not collected uniformly across a field, this is the case for many infield measurements. Such spatial sparsity generates holes in the coverage of data over a field and as demonstrated in Section 3 of this paper, can either result in a large loss of resolution in the resulting field zones or in some cases, a failure in the convergence of the smoothing algorithms.

Measuring multiple variables across the field will rarely result in the same field locations being measured at each time, which will result in, what we term, colocation data sparsity. Current zoning methods require each location to have a complete set of observations. Thus, colocation sparsity can result in a large loss of data, since any location for which only a subset of measurements were observed are omitted from the analysis, compounding the issue of spatial sparsity. Previous applications went some way to address this problem by aligning data to a common grid, however, complete coverage of all variables is rare without a prohibitive level of aggregation.

**Fig. 1:**
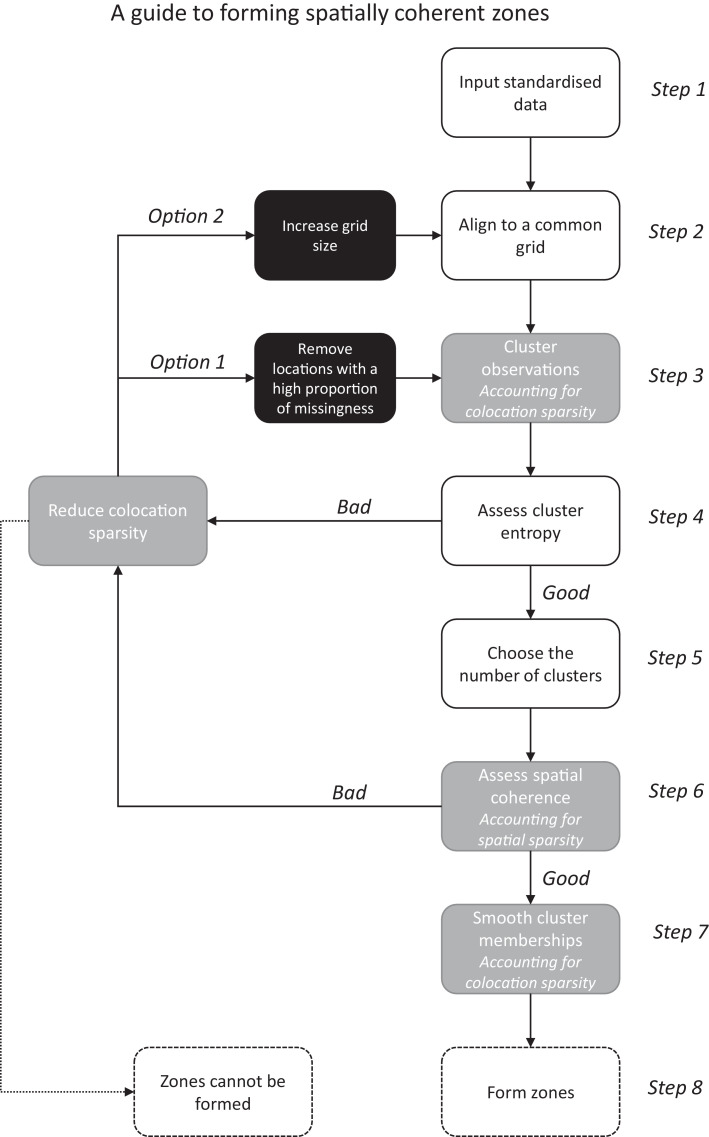
A flow diagram describing the process by which spatially coherent zones are calculated. Boxes highlighted in grey indicate the implementation of our methodological advancements specifically addressing the issues of data sparsity. Boxes highlighted in black indicate additional options one can iterate through to refine the formation of zones under high levels of sparsity.

In this paper we will provide guidance on the formation of spatially coherent zones under data sparsity as summarised in Fig. 1. Specifically, we discuss the issues each type of sparsity creates and describe solutions to these. These methodological advancements are demonstrated through an extensive empirical study of real data collected from multiple fields at different temporal and spatial resolutions. We finish with recommendations of how to form spatially coherent zones under data sparsity and discuss at what point data can be considered too sparse.

## 2 Materials and methods

In the following we describe the three steps taken in [9]; data pre-processing, clustering and smoothing and extend these to account for the issues created by data sparsity.

### 2.1 Data pre-processing

Each variable is first standardised to have unit variance. Since measurements from different variables will rarely co-locate within a field, data are aligned to a regular grid. Where multiple measurements of the same variable align to the same grid location, these are averaged.

The grid size should be chosen carefully. Previous implementations recommended a grid size of 10 m, which produces a reasonable resolution for practical field management. However, the choice of grid size does not only affect the zone resolution but also the zone coherence. A grid size too large, compared to the resolution of the raw data, will result in a high level of spatial averaging and therefore will smooth the data before clustering, something we wish to avoid as detailed in Section 2.3 below. On the other hand, a grid size chosen too small compared to the resolution of the raw data will increase both the spatial sparsity, since not all grid points will have data, and the co-location sparsity, as it will increase the number of grid locations with an incomplete set of measurements. This latter point was particularly important in the original method of Lark as any grid location without a complete set of observations was fully removed from the dataset. As such, choosing too fine a grid, could result in a large loss of data. Since our revised methods allow for locations with an incomplete set of measurements, this is no longer a serious issue.

### 2.2 Clustering

Non-hierarchical methods of clustering have been found to outperform the hierarchical methods on field based measurements due, perhaps in part, to the lack of a hierarchical structure in soil [2]. Furthermore, fuzzy clustering methods enable a good assessment of cluster entropy (see Eq. (3)) and allows one to identify points that lie between clusters, as well as those that are easily classified.

To aid the exposition, we include a description of the original fuzzy c-means algorithm of [10] as follows. Let *z_iv_* be the standardised observation for variable v=1,…,p at location i=1,…,n. The aim of the classification algorithms is to group the *n* objects into *k* classes. Each class q=1,…,k is characterised by a centroid vector ${\bar{z}}_{q}=\left\{{\bar{z}}_{1q},\ldots ,{\bar{z}}_{pq}\right\}$. A fuzzy c-means classification is obtained by minimizing,

(1) $$\begin{array}{c} \sum_{q=1}^{k}\sum_{i=1}^{n}u_{iq}^{\omega }\delta_{iq}^{2}, \end{array}$$

where *u_iq_* is the membership probability of location *i* to class *q* such that $\sum_{q=1}^{k}u_{iq}=1,$ *ω* > 1 is the fuzzification parameter with values close to 1 resulting in a less fuzzy classification (ω=1, returns the non-hierarchical k-means algorithm). As recommended in [2], we set ω=1.25. The variable *δ_iq_* is the vector norm used to measure how well location *i* resembles class *q*. Here, we use the Euclidean norm,

$$\begin{array}{c} \delta_{iq}^{2}=\sum_{v=1}^{p}{\left(z_{iv}-{\bar{z}}_{vq}\right)}^{2}. \end{array}$$

The fuzzy clustering algorithm to minimise (1) is given in Algorithm 1 following the parametrization of [11].

**Algorithm 1:**
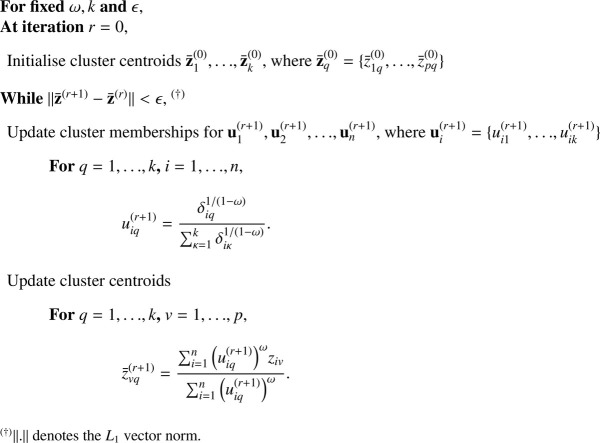
Fuzzy c-means algorithm.

The original fuzzy c-means algorithm can only be applied to the subset of locations for which there is a complete set of observations over all variables. Rather than removing the set of partially observed grid locations, we consider two options. The first runs the fuzzy c-means as above, after which the remaining set of partially observed locations are allocated to each cluster *q* with membership probability,

(2) $$\begin{array}{c} u_{iq}=\frac{D_{iq}^{1/(1-\omega )}}{\sum_{\kappa =1}^{k}D_{i\kappa }^{1/(1-\omega )}}, \end{array}$$

where *D_iq_* is the partial distance given by,

$$\begin{array}{c} D_{iq}=\frac{p}{I_{i}}\sum_{v=1}^{p}{\left(z_{iv}-{\bar{z}}_{vq}\right)}^{2}I_{iv}, \end{array}$$

where *I_iv_* is the indicator function for *z_iv_* observed and $I_{i}=\sum_{v=1}^{p}I_{iv}$.

A second option is to explicitly include the partially observed locations in the optimisation algorithm so that both the membership probabilities and the cluster centroids are optimised with respect to all available data. [11] compared three methods of fuzzy c-means with incomplete data. The best performing algorithm was found to be the optimal completion strategy (OCS) which optimises over the unobserved data via an EM-type algorithm (Expectation-Maximization) and is described in Algorithm 2.

**Algorithm 2:**
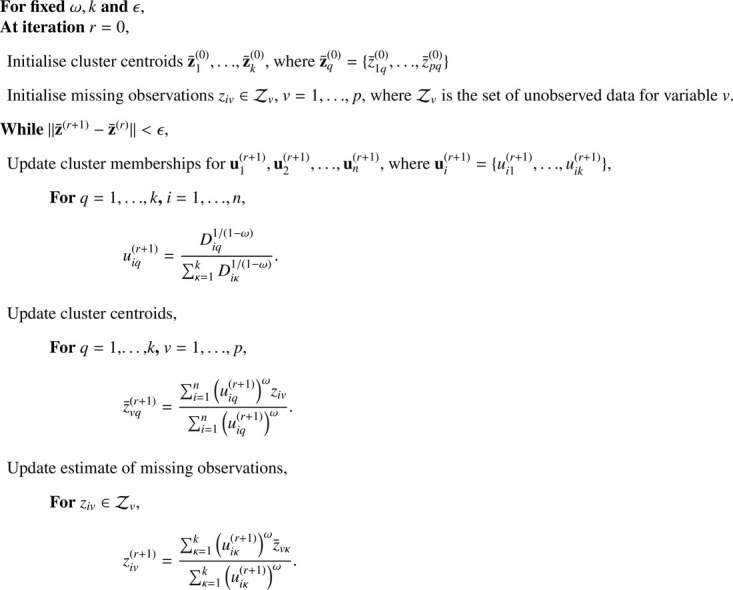
Fuzzy c-means algorithm with optimal completion strategy.

Both methods result in a vector of membership probabilities *u_iq_* for each class, however, choosing the appropriate number of clusters remains a subjective decision. Here, we used the normalized classification entropy, *ξ*(*k*), [12], to identify the most appropriate number of clusters *k*,

(3) $$\begin{array}{c} \xi \left(k\right)=\frac{-1}{\log k}\sum_{q=1}^{k}\sum_{i=1}^{n}\frac{1}{n}u_{iq}\log u_{iq}. \end{array}$$

Following [13], we look for the point, *k*, that falls below the overall trend, such as a local minimum, or the point at which the entropy changes gradient. Note, in the following, we present graphs of 1−ξ, as this scale typically enabled an easier identification of the change points in *xi*.

### 2.3 Spatial smoothing

In this work, we maintain the recommendation that smoothing should happen after the classification or clustering step. Two reasons to do so are, firstly, classifying after spatial smoothing or kriging does not guarantee the spatial coherence of the resulting clusters. Specifically, with a view to on-farm management strategies, we aim to force spatial coherence since the identification of many disjointed zones would be of little practical use in field. Secondly, to smooth the data first, would be to interpolate across the field with the potential effect of artificially increasing the number of completely observed locations. By smoothing in the final step of the zoning process, we avoid the need to propagate imputed data (and their associated uncertainty) through the cluster algorithms.

Following [9], spatial coherence is imposed over the clusters by recalculating the class memberships at each location as a weighted average of the neighbourhood of class memberships. Since membership probabilities form a composition (constrained to sum to 1), this weighted average is calculated after a symmetric log-transformation of the membership probabilities [14],

$$\begin{array}{c} {\tilde{u}}_{\mathrm{iq}}^{*}=\sum_{j\in R}w\left(i,j\right){\tilde{u}}_{\mathrm{jq}}, \end{array}$$

where ${\tilde{u}}_{iq}$ is the transformed membership probability for location *i*, class *q, R* defines the radius of a circular neighbourhood of location *i* and *w* is a weight defined by the dependence between locations *i* and *j*.

The weights *w*(*i, j*) are formed so that points close to location *i* are given higher weight than locations further away and are derived from the variogram function [15],

$$\begin{array}{c} \gamma \left(h\right)=c_{0}+cf\left(h\right), \end{array}$$

where *γ*, termed the semi-variance, is a function of the expected mean squared difference between random variables at locations separated by a distance *h*. The variogram therefore characterises the spatial dependence between points and is incorporated into the weighted smoothing through the following [9],

(4) $$\begin{array}{c} w\left(i,j\right)=\frac{1-f\left(h_{\mathrm{ij}}\right)}{\sum_{l\in R}1-f\left(h_{\mathrm{il}}\right)}, \end{array}$$

where *h_ij_* is the distance between points *i* and *j*.

**Fig. 2:**
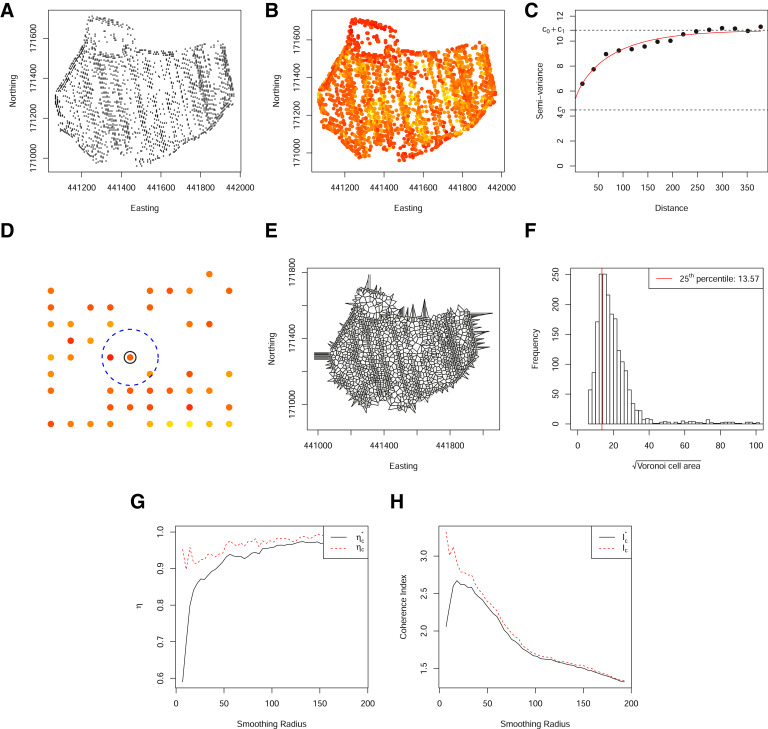
(A) Locations of the set of complete observations for a single field on a grid size of 5 m. (B) Locations are coloured according to the transformed membership probabilities for the most commonly occurring class resulting from a fuzzy c-means clustering with 4 clusters and (C) shows the associated variogram. (D) An illustration of the neighbourhood under spatial sparsity. (E) The Voronoi grid of observed spatial locations. (F) Histogram of the length of Voronoi cell size, calculated as the square root of the Voronoi cell area. (G) The numerator of the coherence index calculated based on a grid neighbourhood (dashed) and a Voronoi neighbourhood (solid). (H) The coherence index calculated based on a grid neighbourhood (dashed) and a Voronoi neighbourhood (solid). (For interpretation of the references to color in this figure legend, the reader is referred to the web version of this article.)

An example variogram is shown in Fig. 2 and illustrates (i) the nugget variance, *c*_0_, which is the spatially independent contribution to the variance, (ii) a period of increasing *γ*, characterising the property that points separated by a small distance *h*, are more similar than points separated by a large distance *h* and (iii) a sill, $c_{0}+c_{1},$ indicating points separated by large distances are spatially independent.

The inclusion of the variogram function in the definition of the weights provides a rational measure of spatial dependence between points. Specifically, [9] and [8] considered models to the multivariate variogram, whilst [7] considered models to the variogram of the first principal component of the data. In these papers, the empirical variogram gives a description of the general spatial structure across all variables. However, the former approach is restricted to the set of complete observations, meaning either all partial observations are removed (a potentially large loss of data) or data are aggregated which will reduce the resolution at which the variogram can be calculated. In contrast, the latter approach, using the first principal component of the data can incorporate partial observations (through, for example, pairwise deletion or imputation methods [16]) but although the first principal component will provide an overall summary of the data, it is not guaranteed to capture spatial variation. Instead, we propose the variogram is calculated from the transformed class membership probabilities. Although there will be *k* possible variograms, one for each class membership, we find in practice that, with the exception of the nugget, very little difference can be seen in the variograms of the different class memberships. Thus, the empirical variogram is obtained from the transformed membership probabilities of the most commonly occurring class, to which the model variogram is fitted. Since there will be a class membership for every location, including those with incomplete measurements (when implementing the revised cluster algorithms), all locations are included in the calculation of the spatial dependence and moreover the variogram will explicitly capture the spatial dependence of the classification.

Not only is the choice of weights in (4) important, but also the choice of *R*, the radius of smoothing. If *R* is too small, clusters remain fragmented, whereas for large *R*, clusters are oversmoothed. [9], defined a coherence index, *I_c_*, which when maximised, defines a radius that balances out the need to reduce spatial fragmentation and to ensure the resulting smoothed clusters are consistent with the original variables,

(5) $$\begin{array}{c} I_{c}=\frac{\eta_{a}}{\sum_{q=1}^{k}\psi_{q}^{2}}. \end{array}$$

Here *η_a_* is the proportion of pairs of points within a distance $a=g\sqrt{2},$ where *g* is the underlying grid size, that belong to the same class and *ψ_q_* is the proportion of units that belong to class *q* (Fig. 2). Such a coherence index maximises the probability that two individuals separated by a distance *a* are in the same class, normalized by the probability that two randomly selected individuals from the dataset belong to the same class. For complete data, the above coherence index works well, however, when data are spatially sparse, this function often has discontinuities making it difficult to optimise. This can be understood through the definition of a coherent neighbourhood. Eq. (5) does this based on the neighbourhood of the underlying grid. However, when data are spatially sparse, relatively few points will have a complete neighbourhood, with many points having, potentially, a single neighbour. Thus, at short ranges, the numerator of (5) quickly saturates. To overcome these discontinuities, we instead define $I_{c}^{*}=\eta_{a}^{*}/\sum_{q=1}^{k}\psi_{q}^{2},$ where $\eta_{a}^{*}$ is calculated using a distance of a* such that a* is the 25th percentile of the square root of the Voronoi cell area, where the Voronoi grid is defined by the Delaunay triangulation of the locations within the field. Defining a neighbourhood based on the observed Voronoi grid ensures a reasonable coverage and a consistent coherence index (Fig. 2).

We also note here, that although in practice the numerator of the coherence index has the largest influence, the denominator is minimised when clusters are of equal size. This may not, in itself, be a necessary property of the resulting clusters and as such, can be downweighted further by raising *ψ_q_* to a higher power.

## 3 Results

In this section, we describe the results from an extensive empirical study designed to investigate how the above methods address the issues created by different types of data sparsity. Specifically, we have three fields with wheat yield measurements obtained from multiple years at a reasonable spatial density. We studied the effects of variable sparsity, by restricting data to different subsets of years, and the effects of spatial sparsity, by considering different grid sizes, on clustering and smoothing. In combination, these enabled us to investigate the effect of colocation sparsity. A summary of these scenarios is given in Table 1. To each data scenario we implemented 3 cluster options,

1. Original fuzzy c-means, requiring complete observations
2. Original fuzzy c-means of complete observations with partially observed locations allocated post-hoc to the most probable cluster (Eq. (2)).
3. Fuzzy c-means with optimal completion strategy

and two smoothing options,

1. Over a neighbourhood defined using the underlying grid alignment
2. Over a neighbourhood defined using the Voronoi tessellation.

**Fig. 3:**
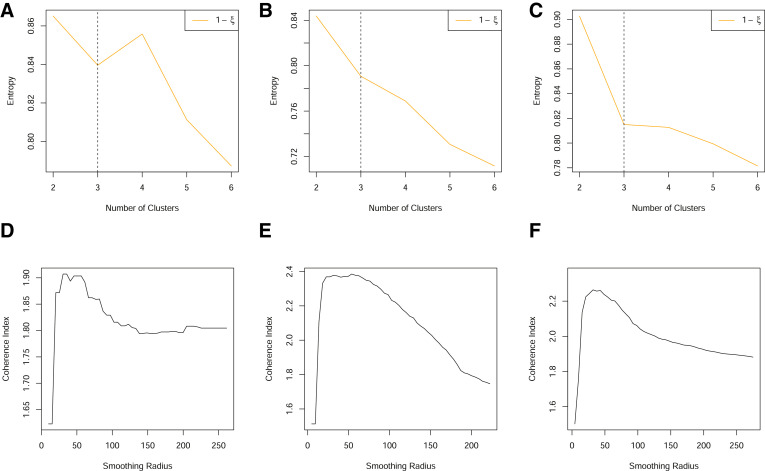
(A)–(C) The relationship between the cluster entropy, *ξ*, and number of clusters. These are illustrative examples of a “bad” (no distinct change point in the gradient of entropy can be identified), “moderate” and “good” (a distinctive change in gradient can be identified) cluster assessment, respectively. (D)–(E) The coherence index plotted as a function of the smoothing radius. These are illustrative examples of a “bad” (jagged, ill-behaved curve), “moderate” and “good” (smooth, with clear maximum identifiable) smoothing assessments, respectively.

For these data, explicit information, such as soil maps, that designate a definitive clustering are not available. As such, no true validation datasets exist that can be used to calculate algorithm error. Thus, to assess algorithm performance, a subjective assessment of the clustering and smoothing was made for each data scenario. The clustering was categorised as “good” if a classification could be clearly identified from the calculated cluster entropy, “moderate” if a classification could be identified, albeit with some sceptism or “bad” if no clear classification could be identified. The smoothing was categorised as “good” if a clear maximum could be identified from the coherence index, “moderate” if a maximum existed but was not clearly identified, e.g. through discontinuities in the coherence index and “bad” if no clear maximum could be identified. Examples of these categorisations are shown in Fig. 3.

**Fig. 4:**
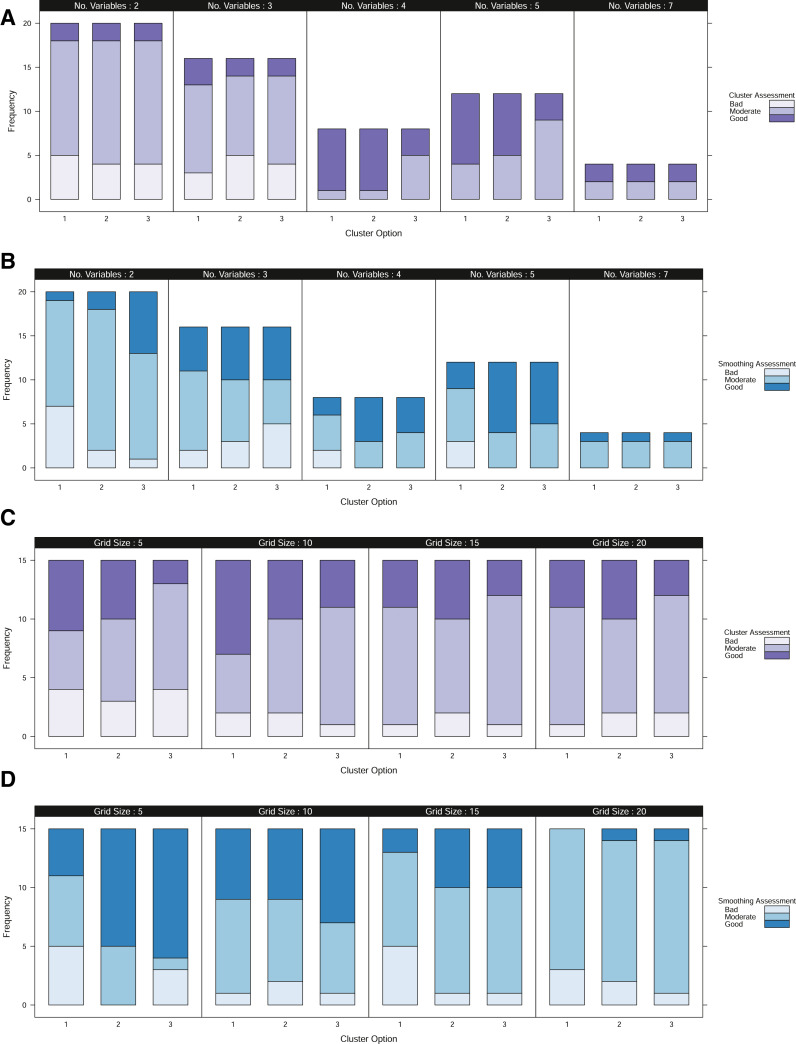
Results from an empirical study of three fields through an assessment of clustering (A) and (C) and smoothing (B) and (D). (A)–(B) The frequency of data scenarios that were considered to have “bad”, “moderate” or “good” assessment for differing numbers of variables (years of data) under each of the three clustering options. (C)–(D) The frequency of data scenarios that were considered to have “bad”, “moderate” or “good” assessment for data aligned to different grid sizes under each of the three clustering options. Frequency, refers to the number of data scenarios of each type see Table 1. Cluster option 1, refers to the original fuzzy c-means, option 2 includes the post-hoc allocation of partially observed locations and option 3 refers to the fuzzy c-means with optimal completion strategy.

The results of this assessment are shown in Fig. 4. From here, a tendency for improved clustering with the inclusion of more variables can be identified (Fig. 4A)). Furthermore at the smallest grid sizes, cluster identification appears to worsen as there is a greatly reduced set of locations which are fully observed (Fig. 4C)). It is interesting to note, that at the smaller grid sizes, the cluster assessment becomes more dichotomous when using the original fuzzy c-means algorithm compared to the two alternative clustering methods. This reflects the fact that the fuzzy c-means relies upon having a sufficient number of completely observed locations to make an effective assessment. In comparison, the alternative approaches incorporate partially observed locations which could both increase available information but also dilute information if there is little overlap in the partially observed subset (e.g. many locations for which only a single variable is observed).

**Table 1:** The number of datasets used for each data scenario.

		Grid size (m)				
		5	10	15	20	
	2	5	5	5	5	20
	3	4	4	4	4	16
Number of variables	4	2	2	2	2	8
	5	3	3	3	3	12
	7	1	1	1	1	4

		15	15	15	15	60

**Fig. 6:**
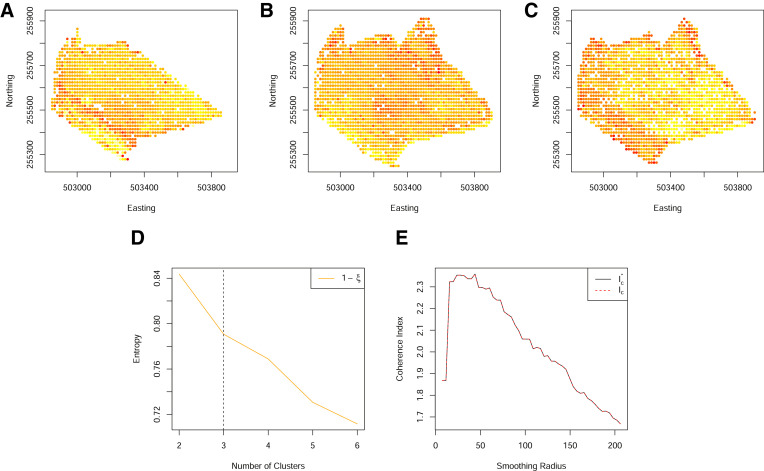
(A)–(C) Standardised yield measurements over three years, aligned to a 10 m grid. (D) The normalized classification entropy of the fuzzy c-means with a nominal selection of 3 clusters. (E) The associated coherence index.

Fig. 4D) shows a tendency for improved smoothing with a finer grid size, particularly when the clustering algorithm allows the inclusion of partially observed locations. In addition, we also find that in scenarios of poorly identified clusters, this coincides with a poorer performance of the coherence index, where peaks are difficult to identify and the coherence index exhibits jagged behaviour (Fig. 6E). This may be due to the relatively little information that distinguishes one location from another, regardless of its position in the field.

## 4 Discussion

### Identifying variable sparsity

**Fig. 5:**
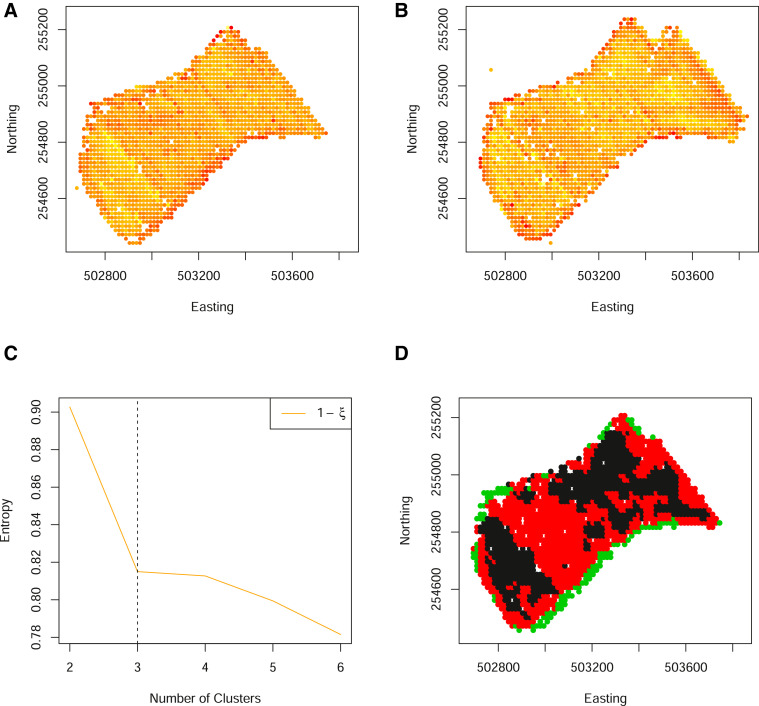
(A)–(B) Standardised yield measurements over two years, aligned to a 10 m grid. (C) The normalized classification entropy of the fuzzy c-means, indicating a choice of 3 clusters is appropriate. (D) The resulting spatially coherent zones (smoothed via the weights of Eq. (4)).

Results shown in Section 3 indicated that cluster identification often improves with the inclusion of more variables. However, distinct zones and clusters can still be formed from just two years worth of data as shown in Fig. 5. Furthermore, the inclusion of more variables does not guarantee cluster formation (Fig. 6).

Thus, before proceeding with the formation of coherent spatial zones, the raw clustering output should be evaluated through an assessment of the cluster entropy (Step 4 in Fig. 1). We have found the minimum number of years required to result in a reasonable clustering (as identified from the entropy) to depend both on the field and the particular subset of years considered. Thus, although there exist recommendations in the literature, (see e.g. [5] for assessment of cotton yields), we recommend a case by case evaluation of the clustering to determine whether resulting zones will be distinct enough to be of use.

### Spatial sparsity impacts coherence and smoothing

**Fig. 7:**
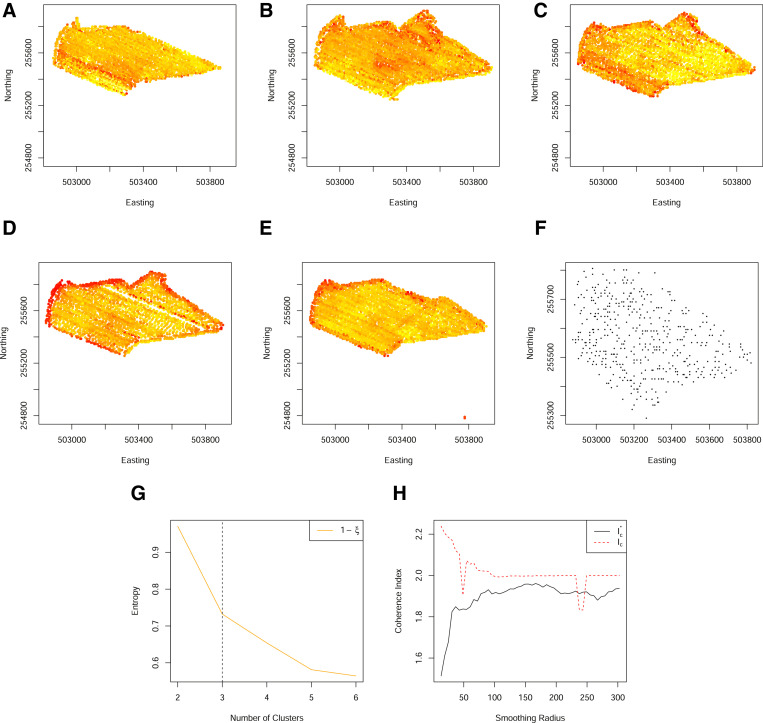
(A)–(E) Standardised yield measurements over five years, aligned to a 5 m grid. (F) The spatial locations of complete observations on a grid of 5 m. (G) The normalized classification entropy of the fuzzy c-means. (H) The associated coherence index based on the underlying grid of 5 m (dashed) and Voronoi cell length (solid). (For interpretation of the references to color in this figure legend, the reader is referred to the web version of this article.)

Fig. 7, shows 5 years of yield data for a single field. When these data are aligned to a 5m grid, there are relatively few locations for which there are a complete set of observations. Despite so few locations with a complete set of observations, clusters can be well-identified. However, due to the spatial sparsity, they cannot be made spatially coherent with the coherence index of Lark. Moreover, although the revised coherence index based on the Voronoi cell size is an improvement, it does not identify an optimal smoothing range (at Step 6 of Fig. 1). In this scenario, data are too sparse to form coherent zones.

**Fig. 8:**
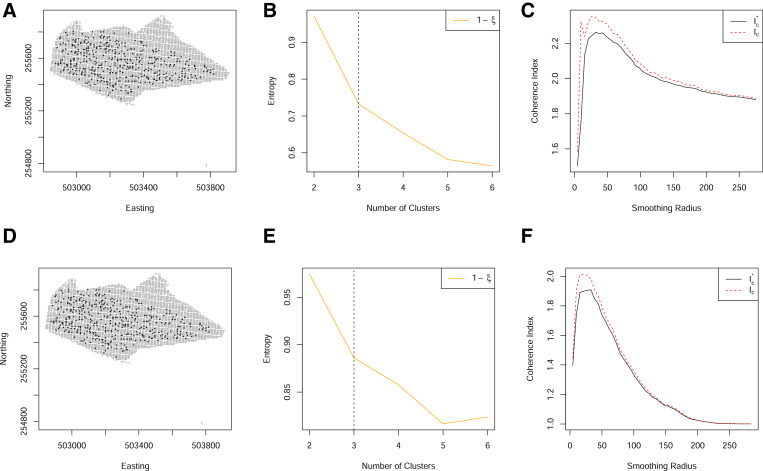
(A) The spatial locations of both complete (black) and partial (grey) observations on a grid of 5 m. (B) The normalized classification entropy of the fuzzy c-means. C) The associated coherence index based on the underlying grid of 5 m (dashed) and Voronoi cell length (solid) using all locations through a post-hoc allocation of to the nearest cluster. (D) The spatial locations of both complete (black) and partial (grey) observations on a grid of 5 m. E) The normalized classification entropy of the OCS fuzzy c-means. (F) The associated coherence index based on the underlying grid of 5 m (dashed) and Voronoi cell length (solid). (For interpretation of the references to color in this figure legend, the reader is referred to the web version of this article.)

Spatial sparsity can be mitigated by retaining the partially observed locations. This can be done through either of the revised clustering algorithms outlined in Section 2.2 (Step 3 of Fig. 1). When implementing the revised clustering algorithms to allow for partially observed locations, a much finer grid of spatially dense data can be used (Fig. 8). The consequence of such is to provide a much improved (smooth and well-defined) coherence index. We find both methods to perform well, although the post-hoc allocation of partial observations will not guarantee clusters to be defined consistently as the cluster centroids are not optimised over the partially observed locations. In implementing the optimal completion strategy, the clustering algorithms required more iterations to converge, and it is sometimes the case that for many locations with partially observed data, may fail to converge. In practice, one may need to consider a combination of variable-wise and unit-wise deletion of observations to reduce the colocation sparsity.

**Fig. 9:**
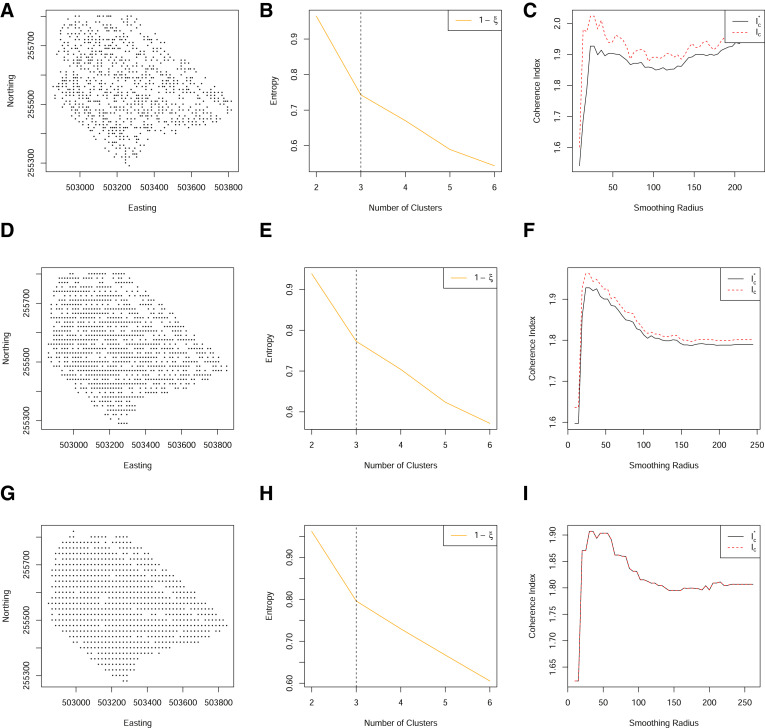
(A) The spatial locations of complete observations on a grid of 10 m. (B) The normalized classification entropy of the fuzzy c-means. (C) The associated coherence index based on the underlying grid of 10 m (dashed) and Voronoi cell length (solid). (D) The spatial locations of complete observations on a grid of 15 m. (E) The normalized classification entropy of the fuzzy c-means. (F) The associated coherence index based on the underlying grid of 15 m (dashed) and Voronoi cell length (solid). (G) The spatial locations of complete observations on a grid of 20 m. (H) The normalized classification entropy of the fuzzy c-means. (I) The associated coherence index based on the underlying grid of 20 m (dashed) and Voronoi cell length (solid). (For interpretation of the references to color in this figure legend, the reader is referred to the web version of this article.)

An alternative solution, is to increase the grid size (Fig. 9). As the grid size increases, the spatial sparsity decreases and the coherence index is better identified. However, for larger grid sizes, the coherence index is less smooth reflecting the higher level of discretization in the grid alignment. Fig. 9 shows a grid size of 15m to be a reasonable compromise between a reduction of spatial sparsity without too much discretization.

### Mitigating data loss from colocation sparsity

**Fig. 10:**
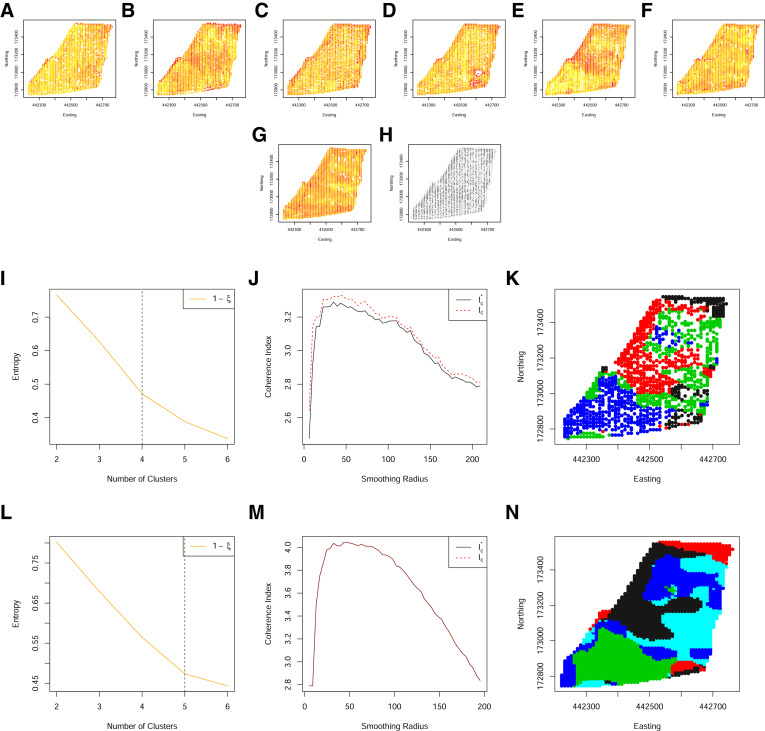
(A)–(G) Standardised yield measurements over 7 years, aligned to a 10 m grid. (H) The spatial locations of both complete (black) and partial (grey) observations on a grid of 10 m. (I) The normalized classification entropy of the fuzzy c-means. (J) The associated coherence index based on the underlying grid of 10 m (dashed) and Voronoi cell length (solid) and (K) the associated smoothed clusters. (L) The normalized classification entropy of the OCS fuzzy c-means. (M) The associated coherence index based on the underlying grid of 10 m (dashed) and Voronoi cell length (solid) and (N) the associated smoothed clusters. (For interpretation of the references to color in this figure legend, the reader is referred to the web version of this article.)

**Fig. 11:**
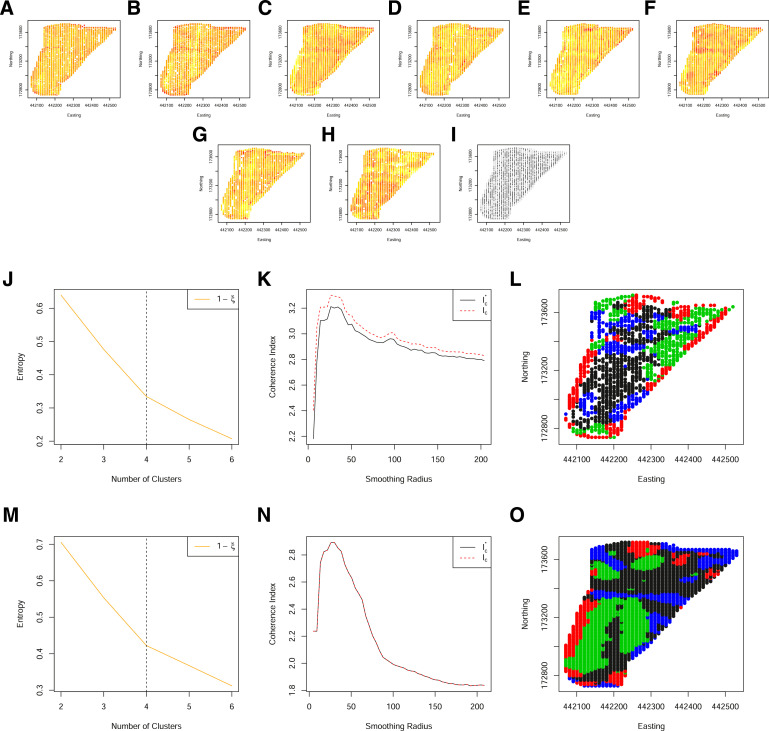
(A)–(H) Standardised yield measurements over 8 years, aligned to a 10 m grid. (I) The spatial locations of both complete (black) and partial (grey) observations on a grid of 10 m. (J) The normalized classification entropy of the fuzzy c-means. (K) The associated coherence index based on the underlying grid of 10 m (dashed) and Voronoi cell length (solid) and (L) the associated smoothed clusters. (M) The normalized classification entropy of the OCS fuzzy c-means. (N) The associated coherence index based on the underlying grid of 10 m (dashed) and Voronoi cell length (solid) and (O) the associated smoothed clusters. (For interpretation of the references to color in this figure legend, the reader is referred to the web version of this article.)

Figs. 10 and 11, demonstrate one of the key advantages to the methodological extensions outlined in Section 2. Specifically, these are two additional fields for which many data are available. In particular, yield measurements have been collected for 7 and 8 years respectively. However, with an increase in the number of years measured, the co-location sparsity increases, resulting in fewer locations having a complete set of observations. The subsequent zones, obtained from the original approach of Lark, although identified, are at a relatively low spatial resolution. It can be seen that allowing for partial observations, increases the spatial resolution of the resulting zones but not at the cost of zone coherency.

## 5 Conclusion

The methodological advances described in Section 2 enable a more efficient use of data by discarding less information in the formation of spatially coherent zones. In particular, we have shown that by extending the clustering methods to cope with partially observed locations, more data are available as input to the coherence index and resulting variogram smoothing. Furthermore, by obtaining a variogram of the transformed class memberships, a complete set of data is available to determine any spatial dependence. However, the membership at each location will not be equally reliable as some will be based on incomplete data. Although this uncertainty is not accounted for explicitly, to a great extent, it will be captured through the class membership probabilities. For example, a location with only a single observation is likely to have a flatter distribution of membership probabilities as it is less clearly associated with a particular cluster profile.

However, despite the advancements described in this paper, a certain level of manual assessment remains a key component. As shown in Fig. 1, an assessment of cluster entropy is required to identify the presence, and associated number, of distinct clusters. Clusters may not be identifiable in the presence of high levels of colocation sparsity (equivalently, in scenarios with a high proportion of locations with an incomplete set of observations). This may be addressed (i) by removing locations with a high proportion of missingness or (ii) by aligning data to a coarser grid. If neither option enables the identification of clusters, more variables are needed to inform the clustering. It is a topic of ongoing work to include alternative data sources, including subjective information, into the methodology in order to better define zones for farm management.

Once clusters have been identified, a second manual assessment can be made of the associated neighbourhood coherence index. This coherence index identifies the range over which to smooth the cluster zones. We have seen that by implementing a Voronoi neighbourhood definition, this coherence index can be more reliably defined under spatial sparsity. However, a manual assessment of the index may still identify a “jagged” behaviour indicative of data that are too spatially sparse. To address this issue, we may consider aligning data to a coarser grid to reduce spatial sparsity at a cost of lower data resolution.

In summary, data sparsity will always be present in one form or another. In this paper, we have investigated the effects of different types of sparsity; variable, spatial and colocation sparsity and how these can be mitigated. In addition, we have provided guidance both on the steps to forming spatially coherent zones and how the use of manual assessments can be used to identify data scenarios that are too sparse to reliably form coherent field zones.

## Declarations of interest

None.
